# Heavy Metal Pollution, Fractionation, and Potential Ecological Risks in Sediments from Lake Chaohu (Eastern China) and the Surrounding Rivers

**DOI:** 10.3390/ijerph121114115

**Published:** 2015-11-06

**Authors:** Lei Zhang, Qianjiahua Liao, Shiguang Shao, Nan Zhang, Qiushi Shen, Cheng Liu

**Affiliations:** 1 State Key Laboratory of Lake Science and Environment, Nanjing Institute of Geography and Limnology, Chinese Academy of Sciences, Nanjing 210008, China; E-Mails: qsshen@niglas.ac.cn (Q.S.); jerrylaw0326@gmail.com (C.L.); 2 Department of Environmental Science, China Pharmaceutical University, Nanjing 211198, China; E-Mails: liaoqianjiahua@163.com (Q.L.); zhangnan920705@163.com (N.Z.); 3 College of Hydrology and Water Resource, Hohai University, Nanjing 210098, China; E-Mail: sgshao@live.cn

**Keywords:** heavy metal, sediment, ecological risk, Lake Chaohu

## Abstract

Heavy metal (Cr, Ni, Cu, Zn, Cd, and Pb) pollution, fractionation, and ecological risks in the sediments of Lake Chaohu (Eastern China), its eleven inflowing rivers and its only outflowing river were studied. An improved BCR (proposed by the European Community Bureau of Reference) sequential extraction procedure was applied to fractionate heavy metals within sediments, a geoaccumulation index was used to assess the extent of heavy metal pollution, and a risk assessment code was applied to evaluate potential ecological risks. Heavy metals in the Shuangqiao and Nanfei Rivers were generally higher than the other studied sites. Of the three Lake Chaohu sites, the highest concentrations were identified in western Chaohu. Heavy metal pollution and ecological risks in the lake’s only outflowing river were similar to those in the eastern region of the lake, to which the river is connected. Heavy metal concentrations occurred in the following order: Cd > Zn > Cu > Pb ≈ Ni ≈ Cr. Cr, Ni, and Cu made up the largest proportion of the residual fraction, while Cd was the most prominent metal in the exchangeable and carbonate-included fraction. Cd posed the greatest potential ecological risk; the heavy metals generally posed risks in the following order: Cd > Zn > Cu > Ni > Pb > Cr.

## 1. Introduction

Worldwide, heavy metals are transported via aerial, terrestrial, and aquatic systems [1]. Because of their persistence in the environment, heavy metals can accumulate in organismal tissues through the food chain. When heavy metals are not metabolized by an organism, especially toxic metals present at low concentrations, they can become toxic to that organism [2,3]. As a result, heavy metal pollution is a significant global problem. The input of heavy metals into aquatic ecosystems is affected by natural processes and anthropogenic activities, including geological weathering, mining effluents, industrial effluents, domestic effluents, urban stormwater runoff, rural nonpoint source pollution, and atmospheric deposition [1,4]. Intensive anthropogenic activities accelerate the input of heavy metals into aquatic ecosystems [5,6]. Heavy metals can be distributed in the water column, suspended particles, and sediments of aquatic ecosystems [7]. Suspended particles adsorb pollutants from the water before being deposited as sediment, resulting in the accumulation of higher levels of heavy metals in sediments [1,8]. In addition, heavy metals may later separate from sediments and reenter the water column because of changes to environmental factors, such as pH, dissolved oxygen, salinity, and sediment disturbances, increasing aquatic organisms’ risks of contamination [9]. 

Total concentration is a sediment indicator that provides information on the status of heavy metal accumulation (contamination); however, this measure does not provide enough information to identify ecological risk or perform a bioavailability assessment [10]. Heavy metals exist in different chemical fractions within sediments, and these different fractions have different levels of mobility, bioavailability, and potential toxicity. As a result, ecological risk and bioavailability are more dependent on the chemical forms of heavy metals within an aquatic environment than on their total concentrations [11,12]. To examine the ecological risk and bioavailability of sediment-based heavy metals, we first had to quantify their fractions. The fractionation of heavy metals within sediment is usually achieved quantitatively using sequential extraction procedures [11,13]. The three-step BCR sequential extraction is a simple procedure that produces a water/acid soluble and exchangeable fraction, a reducible fraction, and an oxidizable fraction. This sequential extraction was first proposed by the European Community Bureau of Reference (BCR) [14] and then thoroughly tested in inter-laboratory studies involving expert European laboratories [15], which verified the extractable contents of the reference material [16]. This procedure was improved in subsequent studies and is widely used in the fractionation of metals from sediment [13]. 

Lake sediment is known to act as a pollutant sink in the water basin [17]. A comprehensive study of the sediments in the lake and surrounding rivers will reveal the transportation patterns of heavy metals from various parts of the basin. Lake Chaohu is a eutrophic lake in Eastern China (N 31°25′–31°43′, E 117°16′–117°5′) with an area of 770 km^2^ and a mean depth of 2.69 m [18,19]. This lake plays a key role in the local fishing industry, the urban drinking water supply, navigation, and tourism activities. Because of rapid industrial and agricultural development and urban expansion around Chaohu, eutrophication, algal blooms, and toxic pollutants in the lake have become serious issues in the past several decades [19,20,21]. Heavy metals in Lake Chaohu have received substantial attention in this century, particularly because of the intensity of the pollution and the importance of the lake. However, although most studies on this topic focus on heavy metals in the lake [22,23,24], no comprehensive study of the heavy metal pollution and ecological risk associated with sediments in the lake and its inflowing and outflowing rivers has been performed. The present study aimed to: (1) investigate heavy metal (Cr, Ni, Cu, Zn, Cd, and Pb) pollution in sediments from Lake Chaohu and surrounding rivers; (2) analyze the fraction distributions and assess the potential ecological risks of heavy metals in the associated aquatic ecosystems; and (3) investigate the influence of river inputs on heavy metal levels in the lake. 

## 2. Materials and Methods

### 2.1. Field Sampling

**Figure 1:**
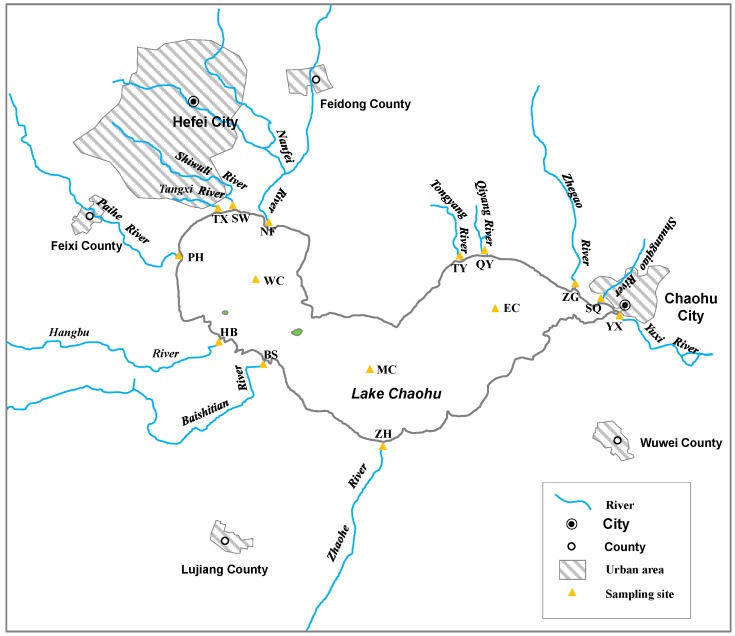
Map of the sampling sites showing 11 rivers flowing into and one river (the Yuxi River) flowing out of Lake Chaohu.

There are 11 main rivers flowing into Lake Chaohu: the Nanfei River (NF), Shiwuli River (SW), Tangxi River (TX), Paihe River (PH), Hangbu River (HB), Baishitian River (BS), Zhaohe River (ZH), Shuangqiao River (SQ), Zhegao River (ZG), Qiyang River (QY), and Tongyang River (TY) (Figure 1). NF, SW, and TX flow through Hefei City, the capital city of Anhui Province, SQ and PH flow through Chaohu City and the county town of Feixi County, respectively. The urban population of Hefei City, Chaohu City, and Feixi Cunty town were separately 2,408,000, 272,000, and 148,000 [25,26]. The major land uses in the drainage areas of these five rivers are constructed land and cropland. The major pollution sources are industrial wastewater, domestic sewage, urban surface runoff, agricultural non-point pollution. For the other six rivers, the drainage areas are mainly cropland, forest, and grassland, with a ratio of about 15:3.6:1 [27]. In these six rivers the major pollution sources are agricultural non-point pollution and rural domestic sewage. The Yuxi River (YX) is the only outflow from Lake Chaohu and discharges into the Yangtze River. We selected 15 sampling sites, three of which were located in Lake Chaohu (western Chaohu (WC), middle Chaohu (MC), and eastern Chaohu (EC)), and the rest of which were placed along the rivers. Each river flowing into or out of Lake Chaohu was sampled at one sampling site. For rivers flowing into Chaohu, the sampling site was located approximately 0.5 km upstream from the river mouth. The sampling site on the outflowing river was located 0.5 km downstream from the river’s starting point. At each site, one sediment core was collected using a core sampler with a Plexiglas tube (length: 50 cm, inner diameter: 8.6 cm) in December 2013. Each tube was sealed with rubber stoppers at both ends and transported to the laboratory immediately after sampling. 

### 2.2. Analyses of Sediment Physico-Chemical Properties and Total Heavy Metals

The 2-cm-deep surface sediment was sliced for each sediment core. Sediment samples were dried at 45 °C to a constant weight, and the weight difference before and after drying was used to calculate the water content (W, %). Dry sediments were ground to pass through 150-μm mesh. Total nitrogen (TN), total phosphorus (TP), and loss on ignition (LOI) of the sediment samples were analyzed. Specifically, TN was examined after sediment digestion by alkaline potassium persulfate, and TP was extracted using 3.5-mol·L^−1^ HCl according to the SMT method [28]. All TP extracts and TN samples were analyzed using a UV-Vis spectrophotometer (UV-2550, Shimadzu, Kyoto, Japan) according to the molybdenum blue and ultraviolet methods, respectively [29]. Organic matter in sediment samples was measured according to the LOI at 550 °C for 5 h. Three replicates were analyzed for each sample site, and the mean results are presented in the Results section. The sediments were completely digested with HCl-HNO_3_-HF-HClO_4_ in a Teflon beaker, and total concentrations of Cr, Ni, Cu, Zn, Cd, and Pb were examined by inductively coupled plasma mass spectroscopy (ICP-MS, Agilent 7700x, Santa Clara, CA, USA).

### 2.3. Sequential Extraction of Heavy Metals from Sediment

Sequential extraction is an important and widely used tool to identify the bound forms of heavy metals in sediment. In this study, an improved three-step BCR sequential extraction procedure was used to separate different forms of heavy metals (Cr, Ni, Cu, Zn, Cd, and Pb) found in the sediment samples [13]. In the first step, sediment samples were extracted with 0.11 mol·L^−1^ acetic acid for 16 h. The resulting substance is called the water/acid soluble and exchangeable fraction and the carbonate-included fraction (F1). In the second step, the residue from step 1 was extracted using 0.5 mol·L^−1^ hydroxylamine hydrochloride, resulting in what is known as the reducible fraction (F2). In the third step, the residue from step 2 was oxidized twice using 8.8 mol·L^−1^ H_2_O_2_ and then extracted with 1.0 mol·L^−1^ ammonium acetate. The resulting fraction is known as the oxidizable fraction (F3). For further details about the extraction procedure, please reference Rauret *et al.* [13]. Similar to the measurement of the total heavy metals present, the residues from step 3 were completely digested with HCl-HNO_3_-HF-HClO_4_ in a Teflon beaker, generating a residual fraction (F4) [22]. The concentrations of Cr, Ni, Cu, Zn, Cd, and Pb in the extraction solution were determined using ICP-MS. The sequential extraction was performed in triplicate, and the means are given in the Results section. 

### 2.4. Calculation and Statistical Analysis

The geoaccumulation index (*I*_geo_) was used to assess the extent of heavy metal pollution in sediments. This index was originally introduced by Müller as follows [30]:

(1) $$I_{geo}=\log_{2}\left(\frac{C_{n}}{1.5\times B_{n}}\right)$$

where *C_n_* is the measured metal concentration *n*, and *B_n_* is the geochemical background metal concentration *n*. In this study, the background value of soils in Anhui Province was used as the *B_n_* (with Cr, Ni, Cu, Zn, Cd, and Pb concentrations equaling 66.5, 29.8, 20.4, 62.0, 0.097, and 26.6 mg·kg^−1^, respectively) [31]. A value of 1.5 was used as the background matrix-correction factor to account for the lithogenic effect. 

Based on the BCR fractionation results, the risk assessment code (RAC) was used to investigate the potential risk attributable to the presence of individual heavy metals. The RAC assessment is classified by the percent of F1 in the total metal. The criteria governing *I*_geo_ and the RAC criteria are summarized in Table 1 [32,33,34].

**Table 1:** Criteria for the accumulation index (*I*_geo_) and risk assessment code (RAC).

*I*_geo_ Classes			RAC Classes		
*I*_geo_	Class	Pollution Status	F1 (%)	Class	Risk
<0	0	unpolluted	<1	1	No risk
0–1	1	unpolluted to moderate	1–10	2	Low risk
1–2	2	moderate	11–30	3	Moderate risk
2–3	3	moderate to heavy	31–50	4	High risk
3–4	4	Heavy	>50	5	Very high risk
4–5	5	Heavy to extreme			
>5	6	extreme			

A correlation analysis was performed to investigate the relationships among the examined heavy metals (Cr, Ni, Cu, Zn, Cd, and Pb) and the physico-chemical properties (W, LOI, TN, and TP) of the sediments. A hierarchical cluster analysis was used to identify the similarity of heavy metals (Cr, Ni, Cu, Zn, Cd, and Pb) in 15 sampling sites following data normalization (range 0 to 1). All statistical analyses were performed using the SPSS 13.0 software (SPSS Inc., Chicago, IL, USA). 

## 3. Results and Discussion

### 3.1. Sediment Characteristics 

In the fifteen examined sites, the sediment water content varied between 47.9% (YX) and 82.7% (SQ) (Table 2). Organic matter (LOI) was the highest at SQ (13.42%) and lowest at HB (4.21%). TP was greater than 1‰ in the NF, SW, PH, and SQ rivers, with the highest value recorded at the NF site (3.09‰). TP was less than 1‰ at other sampling sites, with the lowest value detected at the YX site (0.25‰). TN was less than 1‰ along the HB and YX rivers, while it was greater than 2‰ for the BS, SQ, WC, and EC rivers. At other sites, TN varied between 1‰ and 2‰. 

**Table 2:** Water content (W), loss on ignition (LOI), total nitrogen (TN), and total phosphorus (TP) of sediment samples.

Groups	Sampling Sites	W	LOI	TN *	TP *
		(%)	(%)	(‰)	(‰)
Inflowing rivers	Nanfei River (NF)	63.5	6.44	1.80	3.09
	Shiwuli River (SW)	59.8	6.22	1.65	1.04
	Tangxi River (TX)	59.5	5.24	1.54	0.49
	Paihe River (PH)	53.1	4.38	1.33	1.07
	Hangbu River (HB)	54.6	4.21	0.38	0.52
	Baishitian River (BS)	72.3	7.78	2.62	0.62
	Zhaohe River (ZH)	62.0	4.90	1.32	0.60
	Shuangqiao River (SQ)	82.7	13.42	3.63	2.32
	Zhegao River (ZG)	72.4	7.57	1.88	0.69
	Qiyang River (QY)	63.7	7.07	1.63	0.41
	Tongyang River (TY)	54.6	5.66	1.83	0.63
Lake Chaohu	Western Chaohu (WC)	68.6	8.37	2.39	0.95
	Middle Chaohu (MC)	59.0	6.65	1.82	0.49
	Eastern Chaohu (EC)	68.0	9.93	2.52	0.60
Outflowing river	Yuxi River (YX)	47.9	5.50	0.94	0.25

* TN and TP of the inflowing rivers are derived from Zhang *et al.* 2015 [35].

Of the eleven rivers flowing into Chaohu, higher organic matter content and nutrient loading were present in the NF and SQ rivers [36], which explains the higher TN and TP values in the NF and SQ rivers and the higher LOI in SQ. Although water flow is the highest in HB (15 × 10^8^ m^3^·a^−1^) of all the inflowing rivers, the river has low organic matter content and nutrient loading [36], resulting in relatively lower LOI, TN, and TP values in the sediment from this river. YX is a shipping channel that connects Lake Chaohu to the Yangtze River. The surface sediment in YX has been dredged intermittently to meet shipping demands, resulting in relatively low observed TP, TN, water content, and LOI values in the sediment from this lone outflowing river. Of the three sites located in Chaohu, water content, LOI, TP, and TN were the lowest at MC, while higher values were detected at the WC and EC sites. This is because large amounts of pollutants from Hefei City and Feixi County have been discharged into WC through the NF, SW, TX, and PH rivers, resulting in serious eutrophication and harmful algae blooms in western Chaohu [35,37]. 

### 3.2. Heavy Metals in the Sediments

**Figure 2:**
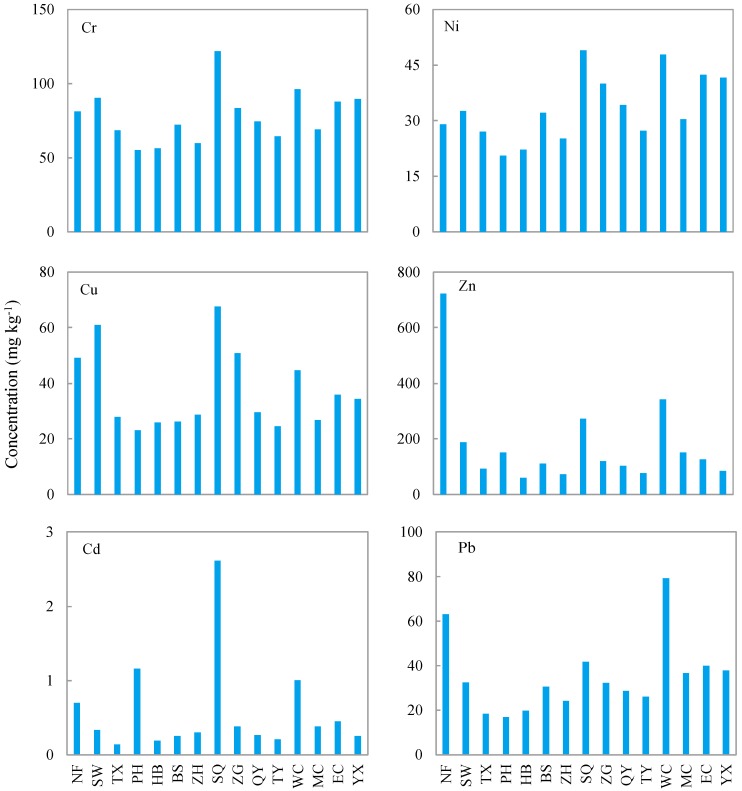
Cr, Ni, Cu, Zn, Cd, and Pb concentrations in sediments from 15 sampling sites. Specifically, there are 11 samples (NF, SW, TX, PH, HB, BS, ZH, SQ, ZG, QY, and TY) corresponding to 11 rivers flowing into Lake Chaohu, 3 samples (WC, MC, and EC) from Chaohu, and 1 sample (YX) from the river flowing out of Chaohu.

Cr, Ni, and Cu had similar distribution patterns throughout the fifteen sampling sites (Figure 2). Cr, Ni, and Cu ranged from 55.2 to 122 mg·kg^−1^, 20.6 to 49.0 mg·kg^−1^, and 23.1 to 67.7 mg·kg^−1^, respectively. The highest concentrations of Cr, Ni, and Cu were identified in SQ, and the lowest were found in PH. The highest concentration of Zn was observed in NF (722 mg·kg^−1^) and was 12 times greater than the lowest value, which was recorded in HB (59.4 mg·kg^−1^). Pb concentrations varied between 17.0 and 79.2 mg·kg^−1^, with the largest and smallest concentrations identified in WC and PH, respectively. The concentration of Cd in SQ (2.62 mg·kg^−1^) was more than 20 times that found at the TX site (0.13 mg·kg^−1^). Cd showed the largest rangeability of the six metals examined across the fifteen sampling sites.

**Figure 3:**
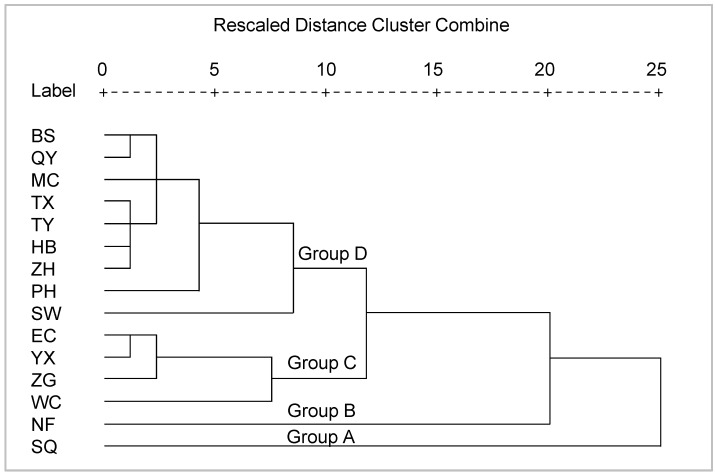
Dendrogram derived from the hierarchical cluster analysis of heavy metal concentrations in the analyzed sediments.

The hierarchical cluster analysis results were rendered as a dendrogram (Figure 3). SQ and NF were separately clustered as one group because of the high levels of heavy metals in these two rivers. WC, ZG, YX, and EC were clustered as a third group and exhibited heavy metal concentrations that were generally lower than those found in SQ and NF but higher than those at other sites. The remaining sites were clustered as a fourth group, in which the presence of heavy metals was generally lower than in the previous three groups.

Of the 11 rivers flowing into Chaohu, the heavy metal concentrations in SQ and NF were generally higher than those in the other studied rivers (Figure 2 and Figure 3). Cheng *et al.* also found that Cr, Cu, Cd, and Pb in NF sediments were higher than in five other studied rivers flowing into Chaohu [38]. This is because both SQ and NF flow through cities: SQ through Chaohu City and NF through Hefei City (Figure 1). The urban populations in Hefei City and Chaohu City were separately 2,408,000 and 272,000 in 2013 [25,26]. Water in the SQ River comes from the Western Flood Drainage Channel of Chaohu City, farm drainage, and surface runoff. The Western Flood Drainage Channel receives industrial wastewater and domestic sewage from Chaohu City and discharges directly into the SQ River; in addition, agricultural nonpoint source pollutants also enter the SQ River as runoff [39]. Large quantities of heavy metals have accumulated in the sediments from the urban rivers of Chaohu City because of anthropological activities [40]. As a result, greater heavy metal levels were recorded in SQ. The NF River receives approximately 1.8 × 10^8^ tons of domestic sewage and industrial wastewater from Hefei City [41], which has resulted in heavy metal accumulation in the sediments of this river [42]. 

Of the three sites in Lake Chaohu, all six heavy metals were present in their highest concentrations at the WC site. The lowest Zn values were recorded in EC, while the concentrations of the other five heavy metals were lowest in MC (Figure 2). Wen *et al.* and Kong *et al.* also found higher heavy metal levels in western Chaohu [43,44]. The discharge of NF is responsible for the higher heavy metal concentrations in this area [43,44]. The heavy metal concentrations in the only outflowing river (YX) were similar to those in EC, to which it is connected (Figure 3). 

In the correlation analysis of heavy metals, Cr was correlated with all other metals except Zn, while Ni was correlated with Cr, Cu, and Pb. Cu was correlated with Cr, Ni, and Cd (Table 3). Zn was only correlated with Pb. Cd was correlated with Cr and Cu. Pb was correlated with Cr, Ni, and Zn. All heavy metals except Zn were significantly correlated with LOI. Cr, Ni, Cu, and Cd were significantly correlated with TN, and Cu, Zn, and Cd were significantly correlated with TP. All of these correlations illustrate that heavy metal pollution is positively related to organic matter pollution in the sediments from the studied area. 

**Table 3:** Pearson correlation matrix for metal concentrations, W, LOI, TP, and TN.

	Cr	Ni	Cu	Zn	Cd	Pb	W	TP	TN	LOI
Cr	1									
Ni	0.905 **	1								
Cu	0.853 **	0.634 *	1							
Zn	0.364	0.172	0.496	1						
Cd	0.644 **	0.467	0.589*	0.372	1					
Pb	0.594 *	0.631 *	0.489	0.738 **	0.316	1				
W	0.633 *	0.604 *	0.589*	0.262	0.566 *	0.360	1			
TP	0.442	0.142	0.620*	0.887 **	0.635 *	0.491	0.407	1		
TN	0.709 **	0.662 **	0.526*	0.283	0.649 **	0.425	0.856 **	0.419	1	
LOI	0.867 **	0.839 **	0.642 **	0.228	0.692 **	0.450	0.831 **	0.376	0.900 **	1

** Correlation is significant at the 0.01 level (2-tailed). * Correlation is significant at the 0.05 level (2-tailed).

### 3.3. Heavy Metal Pollution 

**Figure 4:**
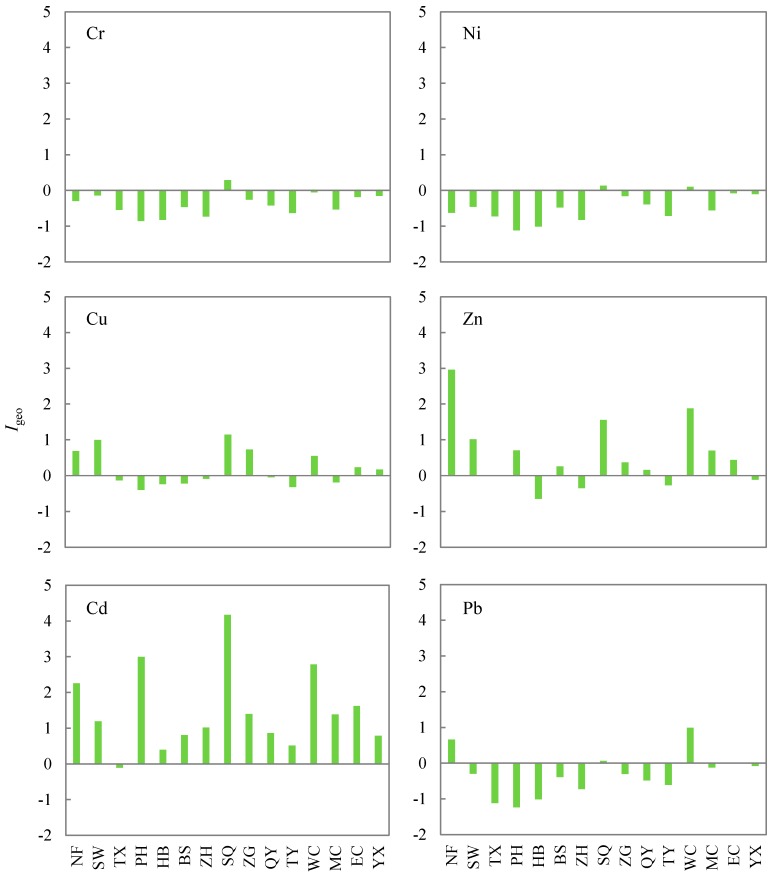
Geoaccumulation index (*I*_geo_) in sediments from 15 sampling sites.

*I*_geo_ relates the accumulation of heavy metals in sediment to background values. Comparing the calculation results (Figure 4) with the *I*_geo_ criteria (Table 1) revealed that, except unpolluted to moderate pollution of Cr in SQ, Ni in SQ and WC, and Pb in NF and WC, these three metals did not exist at contamination levels at any other sites. The *I*_geo_ of Cu in SQ indicated moderate Cu pollution in this river, while other rivers were unpolluted or had a value ranging from unpolluted to moderate pollution for Cu. NF was moderately to heavily polluted by Zn, while SW, SQ, and WC were moderately polluted by this metal. Zn pollution at other sites was unpolluted or ranged from unpolluted to moderately polluted. There was only one site at which Cd pollution was not present (TX) and five sites (HB, BS, QY, TY, and YX) where Cd levels ranged between unpolluted and moderately polluted. The SW, ZH, ZG, MC, and EC sites were moderately polluted by Cd, while the NF, PH, and WC sites were moderately to heavily polluted. SQ was heavily to extremely polluted with Cd.

Of the six examined heavy metals, the pollution levels occurred as follows: Cd > Zn > Cu > Pb ≈ Ni ≈ Cr (Figure 4). In a previous study on the sediment in Chaohu, Cd and Zn exhibited significant pollution levels, while Cr and Ni showed limited accumulation [45,46]. In 45 lakes along the mid-lower reaches of the Yangtze River, the similar heavy metal pollution orders were also found: Zn > Cu > Pb > Ni > Cr [6]. Of the fifteen examined sites, SQ, NF, and WC were generally more heavily polluted than the other sites (Figure 4). This finding was in accordance with the cluster results (Figure 3). The discharge of industrial wastewater and domestic sewage from Hefei and Chaohu City has caused the accumulation of metals in the NF and SQ rivers. NF is a large river with an annual flow rate of 4.50 × 10^8^ m^3^ [36]; when it flows into western Chaohu, it combines with discharges from SW and PH, resulting in relatively serious pollution in WC. The annual flow rate of SQ (0.41 × 10^8^ m^3^) is less than ten percent that of NF [36], and thus, its influence in EC is not as obvious as that of NF in WC. 

### 3.4. Heavy Metal Fractions

**Figure 5:**
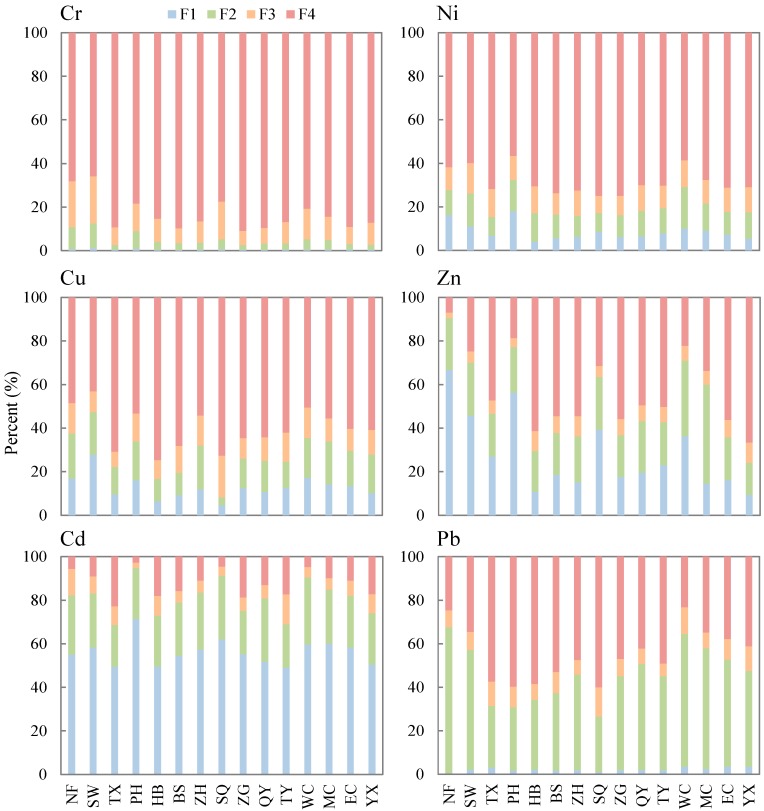
Percentages of Cr, Ni, Cu, Zn, Cd, and Pb existing as different fractions of the BCR sequential extraction procedure for sediments sampled at 15 sites. F1, exchangeable and carbonate included fraction; F2, reducible fraction; F3, oxidizable fraction; and F4, residual fraction.

Cr, Ni, and Cu were primarily present in sediments as F4 (Figure 5), with values ranging from 65.9% to 90.9%, 56.6% to 75.0%, and 43.1% to 74.6%, respectively. For Cr, the orders of the three extractable fractions generally followed an F3 > F2 > F1 pattern, while Ni was present as F3 ≈ F2 > F1. Cu existed generally equally in all three remaining fractions.

Zn exhibited a distinctly different pattern, occurring least often in F3 (2.6%~9.2%), F1 (9.4%~66.6%), F2 (14.7%~45.4%), and F4 (6.9%~66.7%) varied greatly between sampling sites. For example, Zn was present in F1 at relatively higher rates at NF, SW, PH, SQ, and WC than other sites. Similarly, these five sites accumulated higher total Zn concentrations than other sites (Figure 2). In contrast, Zn was present in F2 to a greater extent at WC and MC than other sites, while it existed in F4 in relatively smaller ratios at the six above mentioned sites. 

Pb was present in small percentages in F1 (0.5%~3.4%) and F3 (5.9%~13.4%) and in varying extents in F2 (25.6%~67.1%) and F4 (23.2%~60.1%). The percentages of F2 Pb were less than 40% in TX, PH, HB, BS, and SQ, while the percentages of F4 at these five sites were slightly greater than 50%. For the other ten sites, the opposite situation was observed. 

Cd contributed an average of 56.1% (49.0%~71.3%) of the F1 fraction for all fifteen studied sites. Overall, Cd existed in F1 to a greater extent than any other examined heavy metal. In previous studies on the sediments in Chaohu, the percent values of F1 Cd were reported to be 56% [44] and 63% [45]. In other aquatic ecosystems, Cd was also reported to be present primarily as F1 in sediments; for example, 88% of the Cd in the estuarine sediments from Guadiana saltmarshes on the Iberian Peninsula was reported to be present as F1 [33]. Cd effectively binds to organic matter and carbonate [10]. Cd has been found to exhibit a high affinity for calcium under alkaline and oxidizing conditions, while the selective oxidation of pyrite and the formation of secondary carbonates are capable of retaining Cd [33]. 

### 3.5. Potential Ecological Risk

RAC focuses on the rate at which total heavy metals exist as F1 and classifies potential ecological risks into five classes (Table 1). The potential ecological risks of the six examined heavy metals are shown in Table 4 based on those metals’ F1 percentages (Figure 5) and RAC classes (Table 1). Other than the low risk observed in SW, Cr presented no risk at any other site. Pb resulted in no ecological risks at NF and SQ and presented a low risk for the rest of the sites. The risks presented by Ni were moderate in NF, SW, and PH and low at other sites. Except for the low risk in TX, HB, BS, and SQ, Cu presented a moderate ecological risk throughout the region. The potential ecological risk of Zn ranged from low to very high, with low risk in YX; high risk in SW, SQ, and WC; and very high risk in NF and PH. The potential risks of Cd varied from high (TX, HB, and TY) to very high (other sites). 

Of the six examined heavy metals, the order of potential ecological risk generally followed the pattern Cd > Zn > Cu > Ni > Pb > Cr (Table 4). The potential ecological risks of Cd, Zn, and Cu were in accordance with their overall accumulations in the studied area (Figure 4). The same order was also found in studies concerning the potential ecological risks of heavy metals in Chaohu [22,44]. In other aquatic ecosystems, such as estuaries in Iberian Peninsula and Mahanadi basin (India), there are also higher ecological risks for Cd than other examined heavy metals in the estuarine sediments [10,33]. 

**Table 4:** Potential ecological risk classes based on RAC.

Sampling Sites	Cr	Ni	Cu	Zn	Cd	Pb
NF	1	3	3	5	5	1
SW	2	3	3	4	5	2
TX	1	2	2	3	4	2
PH	1	3	3	5	5	2
HB	1	2	2	3	4	2
BS	1	2	2	3	5	2
ZH	1	2	3	3	5	2
SQ	1	2	2	4	5	1
ZG	1	2	3	3	5	2
QY	1	2	3	3	5	2
TY	1	2	3	3	4	2
WC	1	2	3	4	5	2
MC	1	2	3	3	5	2
EC	1	2	3	3	5	2
YX	1	2	3	2	5	2

Of the eleven rivers flowing into Chaohu, the NF, SW, PH, and SQ rivers exhibited relatively high environmental risks. This is because each of these rivers flows through an urban area, which increases the potential ecological risks associated with some heavy metals. Although intensified agriculture has also been shown to increase heavy metal accumulation in the agricultural region of the Chaohu basin [47], our results demonstrate that generally higher ecological risks occur in rivers flowing through urban areas than in those flowing through rural areas. The ecological risks of heavy metals in YX (flowing out of Chaohu) mirrored those found in EC, in accordance with their similar heavy metal concentrations. Of the three sites in Chaohu, the potential risks of Cr, Ni, Cu, Cd, and Pb were the same across sites. However, there were higher risks associated with Zn in WC than in EC and WC. Clearly, the highest concentration (Figure 2) and potential ecological risk for Zn (Table 4) also existed in NF. In addition, NF flows through Hefei City at a high flow rate, and the discharge of NF plays an important role in the Zn pollution of western Chaohu [22]. The potential ecological risk of Zn in PH was also very high (Table 4). All these likely explain why the potential ecological risk of Zn in WC was high. 

## 4. Conclusions

Of the 15 studied sites, which included 3 in Lake Chaohu, 11 in rivers flowing into the lake and 1 in a river flowing out of it, the *I*_geo_ and cluster analysis showed that the sediments in the NF and SQ rivers were more heavily polluted than those from other sites. This situation may arise because both of these rivers flow through urban areas. In the three lake sites, the heavy metal levels in WC were higher than those in MC and EC, which was contributed by the large quantities of inputs from the NF, SWL, PH, and TX rivers. Of the six examined heavy metals, pollution levels occurred in the following order: Cd > Zn > Cu > Pb ≈ Ni ≈ Cr. As for the fractional constitution, Cr, Ni, and Cu occurred primarily as F4, while Cd made up the largest fraction of F1 of all six examined heavy metals. The order of potential ecological risk generally followed the pattern Cd > Zn > Cu > Ni > Pb > Cr, in which the potential ecological risks of Cd, Zn, and Cu were in accordance with their overall accumulations in the studied area. The potential ecological risk of Cd was high to very high, as was Zn in certain sites. Additional attention should be paid to Cd and Zn and to the NF and SQ rivers in future pollution-control and aquatic environment-restoration efforts in the Lake Chaohu basin. This study also illustrate that rivers flowing through urban may polluted more heavily by heavy metals in a lake basin, and the input of these rivers will cause the heavy metal accumulation and produce the higher ecological risks in the receiving lake area.
